# Challenges in PhD education due to COVID-19 - disrupted supervision or business as usual: a cross-sectional survey of Swedish biomedical sciences graduate students

**DOI:** 10.1186/s12909-021-02727-3

**Published:** 2021-05-22

**Authors:** Emma Börgeson, Matus Sotak, Jamie Kraft, Grace Bagunu, Christina Biörserud, Stephan Lange

**Affiliations:** 1 Institute of Medicine, Department of Molecular and Clinical Medicine, Wallenberg Laboratory and Wallenberg Centre for Molecular and Translational Medicine, 41345 Gothenburg, Sweden; 2 Region Vaestra Goetaland, Department of Clinical Physiology, Sahlgrenska University Hospital, 41345 Gothenburg, Sweden; 3 Revelle College, University of California San Diego, CA-92093 La Jolla, USA; 4 Department of Surgery, Institute of Clinical Sciences, Sahlgrenska Academy, University of Gothenburg, Box 115, 405 30 Gothenburg, Sweden; 5 Department of Surgery, Sahlgrenska University Hospital, 413 45 Gothenburg, Sweden; 6 Department of Medicine, University of California San Diego, CA-92093 La Jolla, USA

**Keywords:** PhD, Graduate program, COVID-19, Biomedical science, Medical sciences training

## Abstract

**Background:**

It remains unclear to what extent the SARS-CoV-2/COVID-19 pandemic disrupted the normal progression of biomedical and medical science graduate programs and if there was a lasting impact on the quality and quantity of supervision of PhD-students. To date, multiple editorials and commentaries indicate the severity of the disruption without providing sufficient evidence with quantifiable data.

**Methods:**

An online survey was submitted to the administrative offices of biomedical and medical PhD-programs at eight major universities in Sweden to gauge the impact of the pandemic on the students. It consisted of multiple-choice and open-ended questions where students could provide examples of positive and/or negative supervision strategies. Open answered questions were coded as either examples of positive or negative support.

**Results:**

PhD students were divided into two groups: those with improved or unchanged supervision during the pandemic (group 1, n = 185), versus those whose supervision worsened (group 2, n = 69). Group 1 received more help from supervisors and more frequent supervision via both online and alternative platforms (email/messages and telephone). There was no significant difference in educational-stage, gender or caretaking responsibilities between the groups.

**Conclusions:**

It is important for the scientific community to learn how to provide the best possible supervision for PhD students during the pandemic. Our data suggests that more frequent supervision, and using a diverse array of meeting platforms is helpful. In addition, it is important for the students to feel that they have their supervisor’s emotional support. Several students also expressed that they would benefit from an extension of their PhD programs due to delays caused by the pandemic.

## Background

The first case of severe acute respiratory syndrome coronavirus 2 (SARS-CoV-2) that causes coronavirus disease 2019 (COVID-19) was reported in December 2019 and the World Health Organization (WHO) declared it a pandemic on March 11, 2020 [1]. The virus has wreaked havoc world-wide, resulting in 83.3 million cases of SARS-CoV-2 and 1.8 million deaths (WHO [2] January 3, 2021: Fig. 1 a), and having devastating socio-economic impact.

**Fig. 1:**
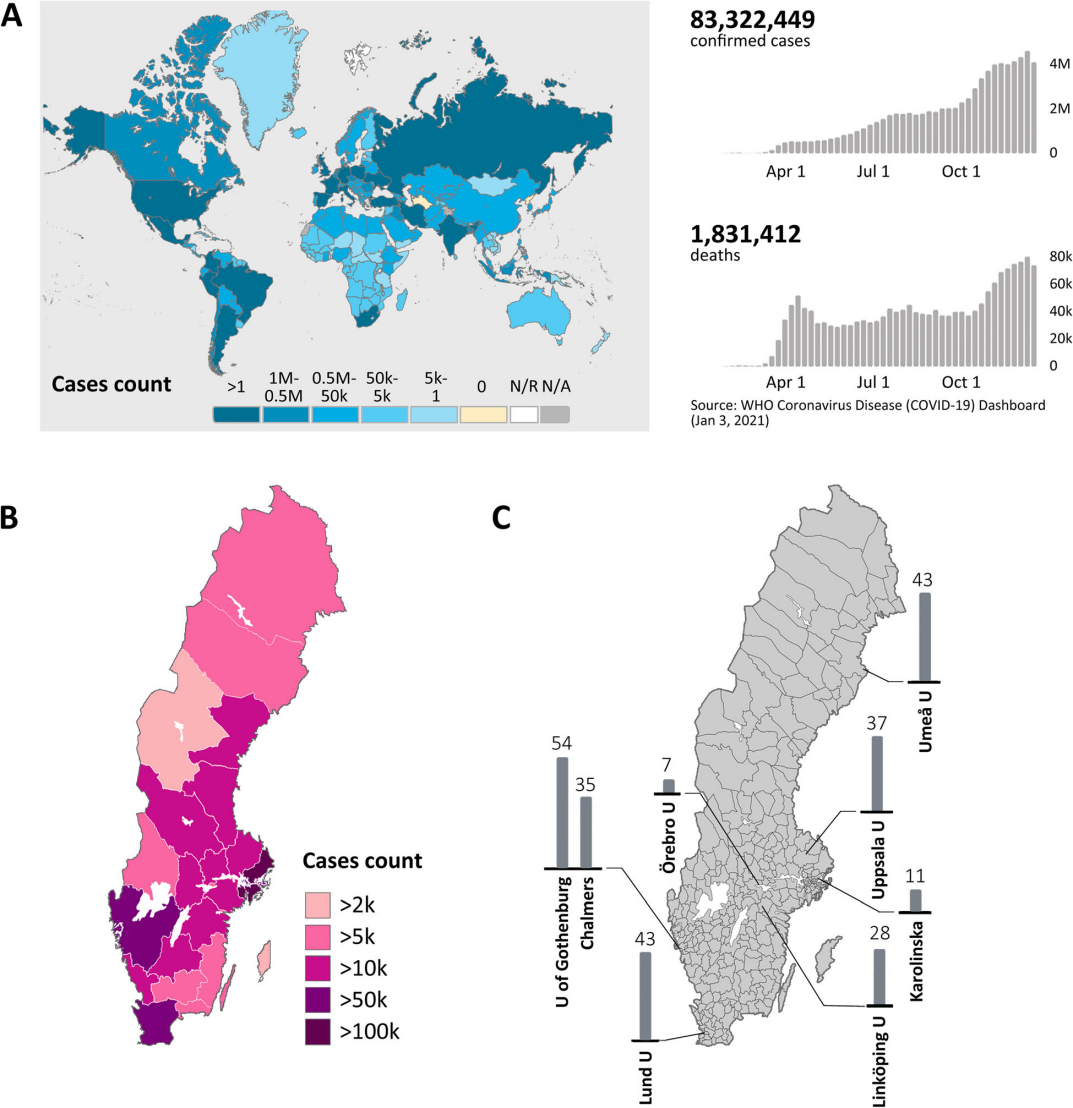
**a**. Number of COVID-19 cases and deaths world-wide, as reported on the World Health Organization COVID-19 dashboard on Jan 3. 2021. **b**. Number of COVID-19 cases in Sweden, shown by region. **c**. Geographic location of universities and number of survey participants

As recently highlighted in several commentaries and editorial articles, the COVID-19 pandemic poses challenges for PhD students and their supervisors [3–8]. In the fields of biomedicine and medicine, the majority of research is conducted through ‘wet-lab’ experiments that need physical presence at the university. Furthermore, students enrolled in medical graduate programs often require access to the hospitals and/or human subjects to conduct their research. Navigating restricted physical access and remote work pose a significant burden on both PhD students and their supervisors.

In Sweden, a PhD in biomedicine or medicine requires 240 educational credits [9] and is typically completed in 4–5 years, although students may work at 50 % pace (e.g. allowing medical doctors to simultaneously work clinically). The PhD is divided into two parts: doctoral courses and an individual research project. The latter should result in authorships on at least four scientific manuscripts, of which one must be accepted for publication. The degree is free of charge, regulated by government decrees, [10] and students are entitled to regular supervision by their primary supervisor.

In this study, we conducted and analysed an online survey with the overarching aim to investigate how the supervision of PhD students has been affected by the pandemic. The survey was conducted in Sweden, where more than 400,000 cases of COVID-19 were reported by the end of 2020 [2, 11] (Fig. 1b). The students answered questions related to their demographics, and provided detailed examples of positive and/or negative aspects of how their supervision has been changed during the pandemic. Our analyses provide suggestions for specific pedagogical approaches for the supervision of doctoral students that are tailored to help them finish their studies and successfully complete their PhD program during this difficult time.

## Methods

### Study design and participants

The survey investigated how the education, supervision and mentoring of PhD students in Sweden has been affected by the COVID-19 pandemic (Table 1) and was conducted using Entergate software ES Maker [12]. It was distributed via the administrative offices of biomedicine and medicine graduate programs at 8 Swedish universities, between November 2020 and March 2021. A total of 262 answers were received (Fig. 1 c).

**Table 1:** Survey cover and questions. Multiple-choice answers are highlighted by square brackets and italicised text

**Demographic and background questions**	1. **What gender are you?***[Female; Male; Do not want to state; Other]* 2. **Do you have caretaking responsibilities?** Caretaking includes childcare, schooling, eldercare, disabled care. *[Yes; No; Prefer not to answer]* 3. **What year in your PhD are you?***[years in program – out of planned years]*
**Pedagogical perspective questions**	1. **Has any of the following changed** [during the pandemic]**?** a) **Quality of the mentoring?***[Improved; unchanged; worsened; N/A]* ***Answers to this question were used to segment respondents into two groups***: *- Group 1 answered that their mentorship had been improved or was unchanged, * *- Group 2 experienced a worsened mentorship during the pandemic.* b) **Frequency of the supervision?***[Improved; unchanged; worsened; N/A]* 2. **During the pandemic, has the format of your meetings with your supervisor(s) changed?***[More; unaffected; less; none; N/A]* 3. Open answered question: **What (if anything) do you wish that the supervisors/mentors would change in order to help you cope with the COVID-19 related restrictions during your PhD?***[Nothing, I did not need any help; I got all the help I needed, specifically my mentor helped me (fill in the box); I did not get help, but would suggest the following (fill in the box)]*
Survey cover and informed consent	The purpose of this survey is to understand how the COVID-19 pandemic has affected PhD students in Sweden, with a specific focus on the doctoral programs that require experimental/medical/biomedical research. By answering this questionnaire, you give your consent that your answers may be analysed and used for educational purposes and/or in a scientific publication. This is an anonymous questionnaire. Please do not provide any personal information (e.g. name, email addresses, phone numbers etc.) in the open answered questions. The program used to generate the questionnaire and collect the data does not register IP addresses. Overall, the answers received will be compiled and analysed on a group level. For the questions where it is possible to provide detailed comments (i.e. open answers), the text may be analysed in isolation. However, all answers will be reported in an anonymised format. This survey is conducted by Dr. Emma Börgeson at the Wallengerg laboratory, Sahlgrenska University Hospital and the University of Gothenburg.

### Ethics

The survey was assessed by the Swedish Ethical Review Authority (Dnr 2021 − 00481) and found to be exempt. For conducting the survey, we followed the general principles and recommendations provided by the Helsinki Declaration [13] and the Belmont Report [14]. Written informed consent to participate in the study was obtained from participants as outlined on the survey cover sheet shown in Table 1.

### Data analysis and availability

Participants were grouped based on how they assessed the changes in the quality of mentorship during the pandemic. 260 of the 262 participants answered the question. Six respondents answering ‘not applicable’ (N/A) were excluded. The remaining 254 respondents were divided into two groups: group 1 contain PhD students that experienced improved or unchanged mentorship, while students in group 2 experienced worsened supervision and mentorship.

The open-ended question was independently analysed using inductive thematic analysis [15]. Briefly, the answers were carefully read after which they were coded, analysed and categorised into examples of positive and negative support (Table 2). When highlighting quotes, obvious spelling mistakes were corrected to facilitate reading. The original dataset is available from the corresponding authors upon reasonable request.

**Table 2:** **Thematic analysis of the question: “***What (if anything) do you wish that the supervisors/mentors would change in order to help you cope with the COVID-19 related restrictions during your PhD?”*. Note that the total % may be higher than 100 %, as a respondents’ complete answer may fit several themes

	Group 1 56 answers		Group 2 30 answers	
**Positive examples of support received during COVID-19**	**Nr of answers**	**% of answers**	**Nr of answers**	**% of answers**
**Theme 1: Good help with the doctoral studies** • Help rescheduling and reorganizing project due to COVID-19 • Flexible meeting times and lab-work duties	**11**	**20 %**	**0**	**0 %**
**Theme 2: Good emotional support** • Received more contact, support, sympathy. • Received support related to mental health. • Supervisor has more time for the student. • Supervisor/student/group has an open dialog on COVID-19. • Supervisor gives advice on how to stay focused when working from home.	**14**	**25 %**	**3**	**10 %**
**Theme 3: Good administrative support** • Supervisor arranges an PhD extension • Supervisor provides administrative help, rearranging courses	**1**	**2 %**	**1**	**3 %**
**Theme 4: Good work environment** • Supervisor arranged ergonomic support in home environment • The student is offered ample opportunities to work from home and/or in a safer environment (e.g. online calendars to avoid crowds, encouraging zoom meetings etc.)	**4**	**7 %**	**1**	**3 %**
**Negative examples highlighting lack of support during the pandemic**				
**Theme 5: Lack of support in doctoral studies** • Requests help re-structuring the PhD in response to COVID-19 • Need of more follow-up meetings and clearer rules	**10**	**18 %**	**4**	**13 %**
**Theme 6: Poor emotional support** • Requests more contact, encouragement and empathy. • Requests support with mental health issues. • Would have liked a better understanding of that is its difficult to work from home, e.g. with kids around. • Requests an open dialog regarding COVID-19.	**9**	**16 %**	**12**	**40 %**
**Theme 7: Lack of administrative support** • Requests PhD extensions • Requests practical advice, e.g. administrative help, rearranging courses	**3**	**5 %**	**8**	**27 %**
**Theme 8: Poor work environment** • Requests ergonomic support in home environment • Requests help adjusting to online work • Requests that journal clubs are available online • Requests that at-home work would be encouraged • Feels pressure to work in the lab with exposure to COVID-19	**7**	**13 %**	**6**	**20 %**

All statistical analyses were done using Prism 8 (GraphPad Software). Answers that had too few respondents to be analysed (e.g. the not-applicable (N/A) option) were excluded from the statistics, as indicated by “ε”. When illustrating results in the figures, each group was set to 100 % to facilitate comprehension.

## Results

### Demographic analysis of study participants

**Fig. 2:**
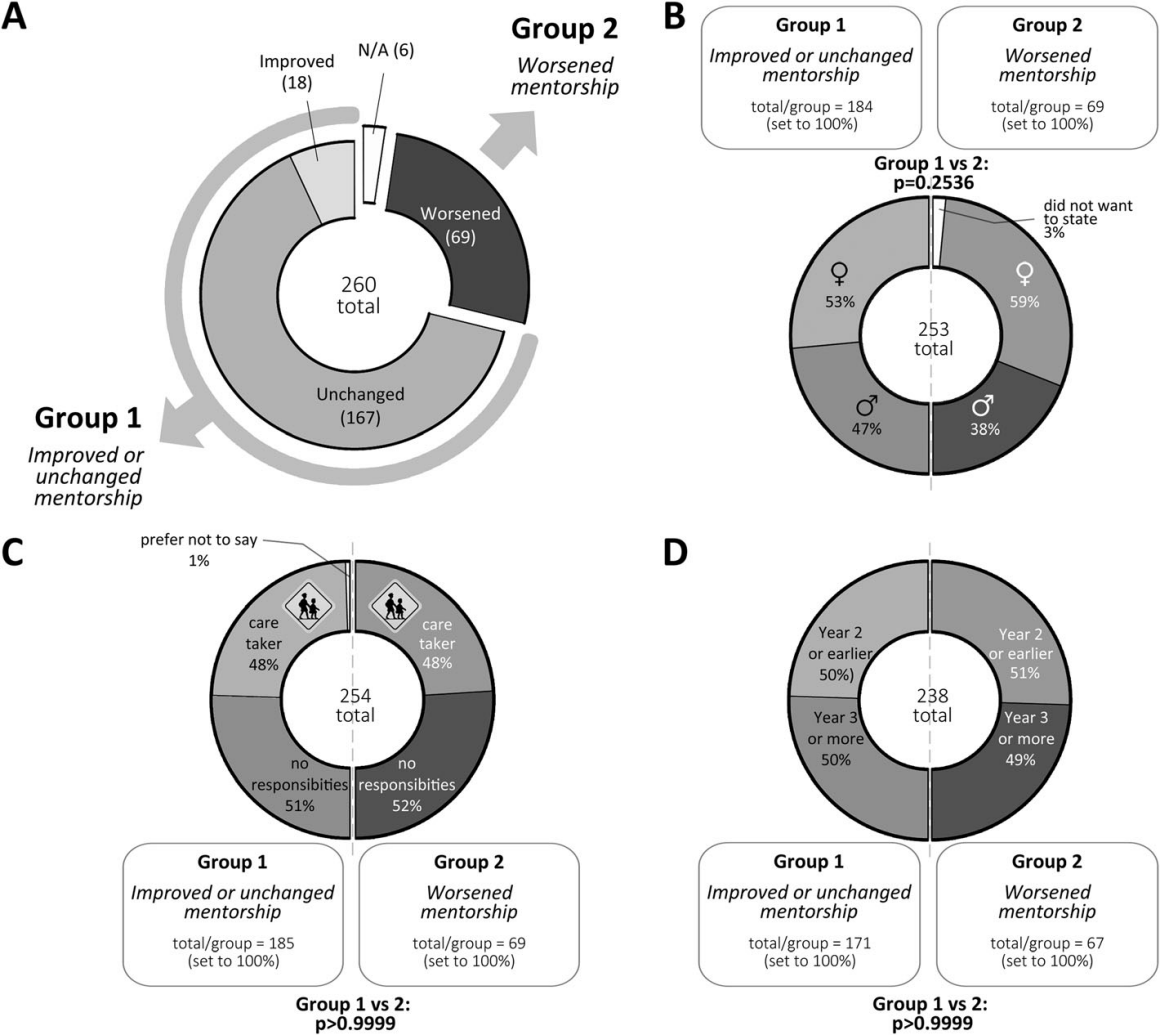
Demography of survey participants. **a**. Grouping of responses to the question whether the quality of the mentoring changed. Respondents in group 1 experienced improved or unchanged mentorship, while respondents in group 2 experienced worsened mentorship during the pandemic. The total number of participants, and numbers of respondents for each group are shown. **b**-**d**. Analysis of participants’ gender distribution (**b**), caretaking responsibilities (e.g. childcare, eldercare, etc.) (**c**) and educational stage (**d**) revealed no statistical differences between groups 1 and 2. The number of total participants for each question, the number of responses and distributions (in %) in each group, as well as p-values determined by comparison between groups 1 vs. 2 using Fisher’s exact test are shown

Study participants were separated into two groups based on whether their quality of mentorship had changed (Table 1). Students in group 1 experienced improved or unchanged mentorship/supervision during COVID-19 (185 students), while group 2 where students that experienced worse mentorship/supervision during the pandemic (69 students) (Fig. 2 a).

The distribution of men and women, as well as levels of caretaking responsibilities, were similar between the groups (Fig. 2b-c). We also evaluated answers according to the educational stage of the PhD students, as students at an earlier career stage (2 years or less) might perceive pandemic-related restrictions differently compared to those at a later stage (after the half-time assessment - later than 2 years). However, there was no difference in early versus late stages between groups 1 and 2 (Fig. 2d).

### Change in supervision format during the COVID-19 pandemic

**Fig. 3:**
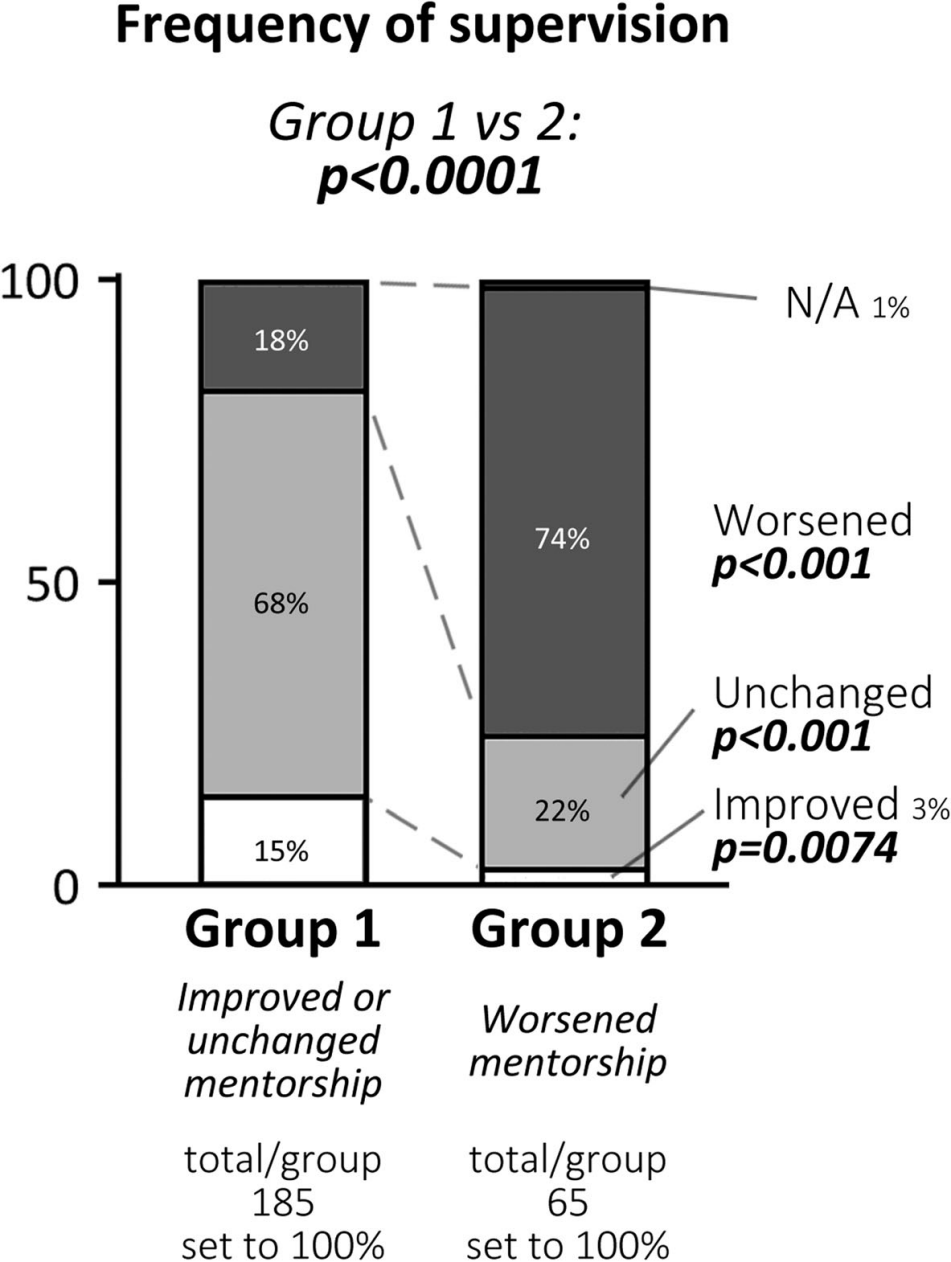
Changes in the supervision frequency during the pandemic. Shown is the number of total participants, the number of responses and distributions (in %) in each group, as well as p-values determined by comparison between groups 1 vs. 2. Significant differences in responses between groups 1 and 2 and specific subgroups were calculated using Fisher’s exact test

The frequency of supervisory meetings was significantly different between groups 1 and 2. Group 1 either had increased (15 %) or unchanged (68 %) number of meetings with their supervisors during the pandemic (Fig. 3). In comparison, nearly 74 % of students in group 2 indicated that the frequency of supervision decreased during the pandemic.

**Fig. 4:**
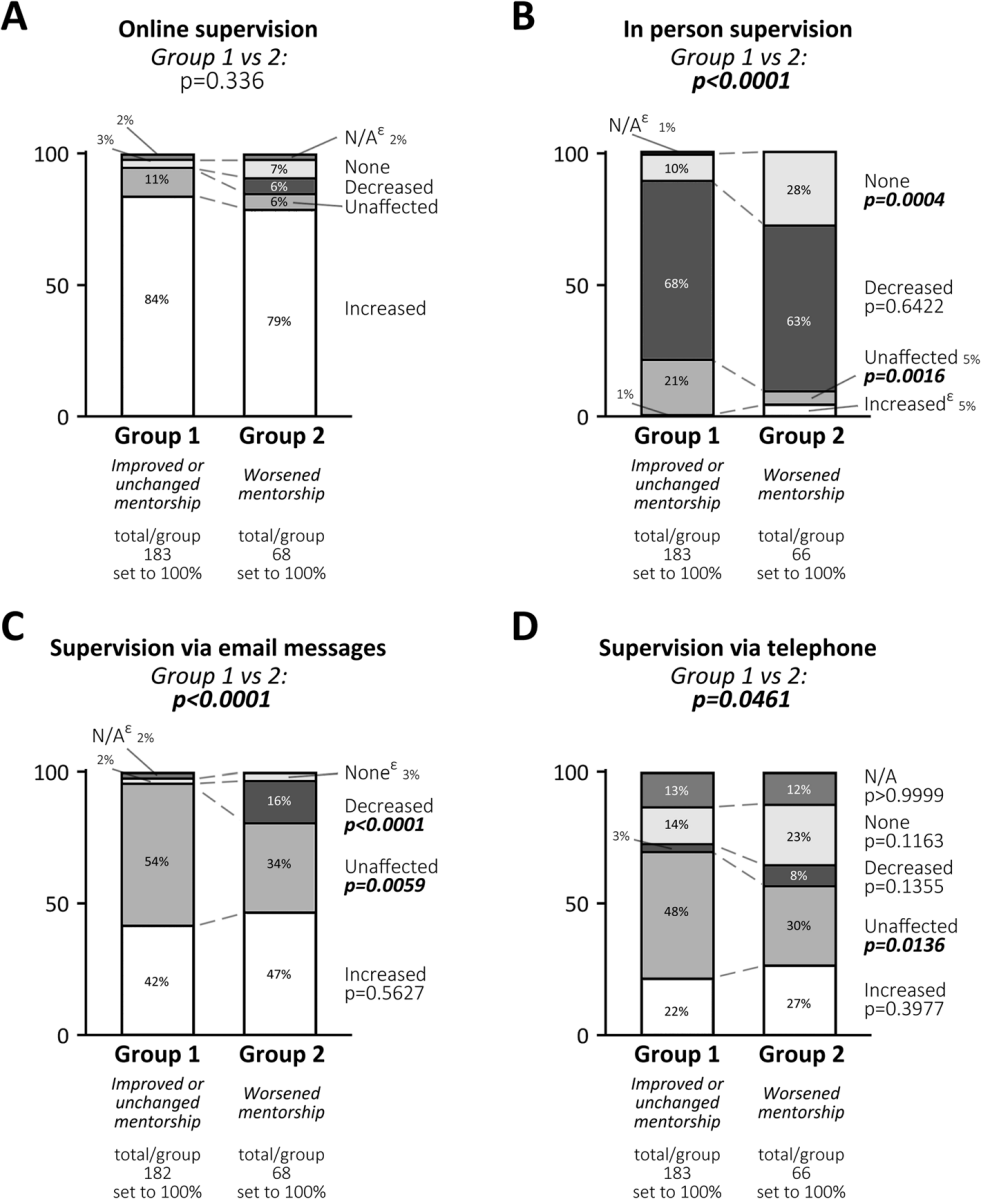
Changes to the supervision done (**a**) online (e.g. via Zoom, FaceTime, Skype), (**b**) in person, (**c**) via email messages or (**d**) via phone. Shown is the number of total participants, the number of responses and distributions (in %) in each group, as well as p-values determined by comparison between groups 1 vs. 2. Significant differences in responses between groups 1 and 2 and specific subgroups were calculated using Fisher’s exact test

The students were subsequently asked how the format of their supervision had changed during the pandemic, and what medium (online, telephone, in-person, etc.) was used for supervision meetings. Both groups 1 and 2 had overall more online meetings, but the increase was similar between the groups, suggesting that group-specific differences are not due to a switch to an online meeting format *per se* (Fig. 4 a). A majority in both groups indicated that in-person meetings decreased (Fig. 4b), but group 2 had a higher fraction of students that had no in-person meetings at all (Group 1:10 %, Group 2: 28 %, *p* = 0.0004), while more students in group 1 reported that in-person meetings were unaffected by the pandemic (group 1: 21 %, group 2: 5 %, *p* = 0.0016). Comparing the methods of communication through email/messages (Fig. 4 c), group 1 had a higher proportion of students where the communication was unchanged (group 1: 54 %, group 2 34 %, *p* = 0.0059), while group 2 received significantly less supervision via emails as compared to before the pandemic (group 1: 2 %, group 2: 16 %, *p* = < 0.0001). There were few differences between groups 1 and 2 when it comes to supervision via telephone (Fig. 4d), although nearly half of students in group 1 (48 %) indicated that use of this medium was unchanged, compared to only 30 % of students in group 2.

### The importance of support provided by supervisors/mentors during the pandemic

**Fig. 5:**
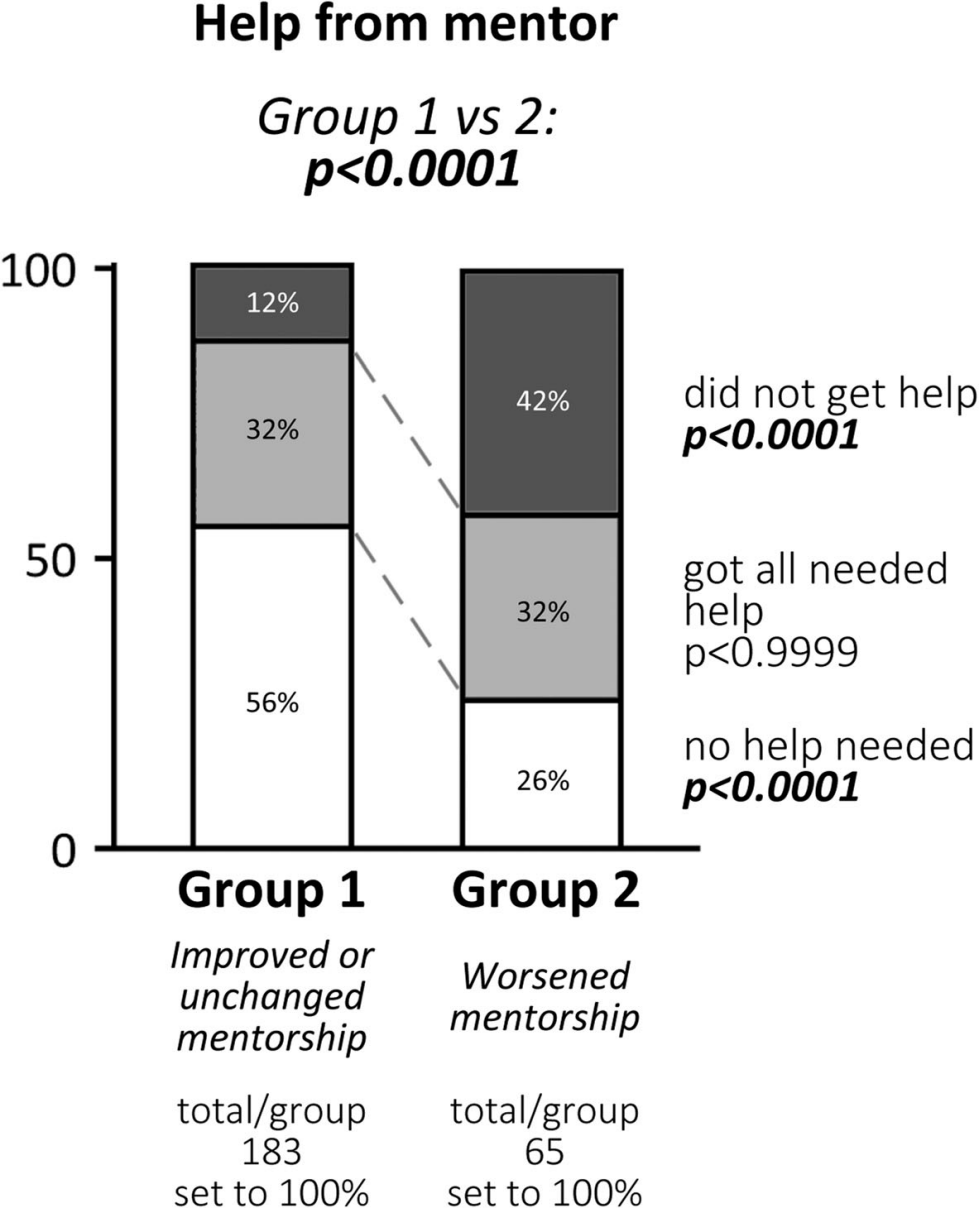
Help received from the supervisor to cope with COVID-19 related restrictions during the pandemic. Shown is the total number of participants, the number of responses and distributions (in %) in each group, as well as p-values determined by comparison between groups 1 vs. 2. Significant differences in responses between groups 1 and 2 and specific subgroups were calculated using Fisher’s exact test

The students were subsequently asked: *“What (if anything) do you wish that the supervisors/mentors would change in order to help you cope with the COVID-19 related restrictions during your PhD?”.* Group 1 received more help from their supervisors. However, students in this group were also more likely to answer that they did not need additional help compared to participants in group 2 (Fig. 5).

We included an open answer box for this question. Participants could describe the additional help they had received during the pandemic, or what support was lacking. In total 86 students (32 %) provided specific answers in the text field. These data were coded as summarised in Table 2 and described below.

#### Positive examples of support received during COVID-19

[**Theme 1: Good help with the doctoral studies**]

A large proportion of students in group 1 (20 %) reported that their mentors have been flexible with meeting times, lab-work, deadlines and rescheduling events when needed. None of the respondents in group 2 gave a comparable answer. Importantly, the students in group 1 reported specific examples of help they received for reorganising their PhD project in relation to COVID-19 imposed restrictions.

*We have done some re-scheduling in prioritised tasks to use the time better [when] things are delayed.*

*Setting up lab meetings and other meetings on zoom, discussing covid-related matters and how to improve the working conditions (especially in the work place). For example, we made an online booking system to schedule lab work so that we are not more than a max number of people working at the same time.*

**[Theme 2: Good emotional support]**

Many students in group 1 (25 %) said they received support from their mentors during the pandemic, with ample opportunities to connect through online meetings, text messages or email. Indeed, some students perceived that their supervisors were less busy and had more time to mentor them during the pandemic. Others reported that it was particularly helpful when mentors gave concrete advice on how to stay focused when working from home. The students also appreciated when supervisors showed sympathy and interest in their mental and physical well-being. A positive open dialogue that allowed for discussions on pandemic-related disruptions was also mentioned.

*Due to less time spent traveling to different meetings, my mentors are more available and have more time for meetings and respond [to] my work.*

*They help me by being supportive, [care] about my wellbeing and [that] of my family abroad.*

*We discussed as a group how to handle it in reference to our own needs and came to a group conclusion about coping with the restrictions.*

*Understanding and checking in. Being flexible regarding the current situation and making it clear that it is not my job to solve it.*

**[Themes 3 and 4: Good administrative support; Good work environment]**

One supervisor arranged an extension for the PhD student, which was appreciated and reduced stress. Students also reported that supervisor provided ergonomic support and arranged opportunities to work from home and/or in a safer environment, e.g. by establishing an online calendar to avoid crowds and encouraging zoom meetings.

#### Negative examples describing lack of support

**[Themes 5 and 6: Lack of support in doctoral studies; Poor emotional support]**

Several students asked for help to restructure their PhD in response to the pandemic, specifically mentioning the need for more follow-ups and clearer rules. However, the majority of students in group 2 belonged to Theme 6 (40 %), expressing the wish to have received more encouragement, contact and empathy, as well as support with mental health issues.

*Acknowledging the difficulties of [the] current situation, including not being able to travel to see family, the pandemic’s impact on the ability to concentrate and perform mental work, considering extensions of study time, asking about the mental health of their employees and offering support, normalising the inability of “performing as usual” under these circumstances.*

*Be more hands-on! Call, message, email [to see] how everything is going!*

**[Themes 7 and 8: Lack of administrative support; Poor work environment**]

Many students stated that they would like help with providing a PhD extension due to pandemic-related delays. Concerning comments were received that students felt pressured to work in the lab regardless of the exposure risk to COVID-19.

*I would like my supervisors to not push me to do experiments that are not necessary or important. Having to do unnecessary experiments makes me stressed as I will have to […] expose myself more to covid-19. I would also like if supervisors cared a bit, [...] apparently there is no possibility of extension, but how about reducing the ‘requirements’? For example instead of having 4 articles maybe these could be reduced to three?*

One student requested support with home ergonomics and a few students requested help adjusting to online work (e.g. attendance of journal clubs should be available online, at-home work should be encouraged).

Finally, one student requested more practical advice, e.g. administrative help to rearrange courses and for the supervisors to be more active in the online meetings.

*All our lab meetings, group seminars and journal clubs have been cancelled since March, I wish they would resume.*

*” Be prepared before meetings. Use the sharing function in meeting. Put on the web cam. …“*.

## Discussion

### Successful supervision strategy during the pandemic: providing practical help and emotional/mental support

One of the main tasks of a PhD supervisor is to help and support the doctoral candidate. Students in group 1 reported that they received more help from their mentors, even though they also answered that they did not need as much help as those in group 2. We interpret these data as that the supervisors in group 1 may have been more active in reaching out and offering help, perhaps even when it was not requested. On the basis of the survey data, we would argue that this is a successful educational approach.

The pandemic has led to changes in the format of the supervision, as meetings moved primarily online. Noteworthy in the results was that although both groups experienced a similar increase in online meetings, group 2 had a reduction in the meeting frequency and received less supervision via alternative platforms (e.g. email or telephone). Students in group 2 highlighted the need to employ several ways of communicating, but specifically suggested to increase the use of online platforms. Interesting, new research indicate that student academic leaning is enhanced when utilizing a multitude of online tools [16–19]. This may be important to note for future recommendations on how to conduct online doctoral supervision, as recently reviewed by Gray and Costa [20].

A positive notion among many students was that they appreciated when their supervisors had ample time for them, providing regular meetings and structured advice. This may be related to a recent study by Wang and DeLaquil [21] that provided a guide to good PhD mentorship during the pandemic. Examples included to schedule regular meetings without set agendas to discuss research in an unstructured environment and promote creativity. Indeed, a handful of students reported *improved* PhD mentorship during the pandemic, mostly due to supervisors having *more* time and providing regular meeting opportunities over a range of different platforms.

 Students also found it important that their supervisors cared about their physical and mental wellbeing. Studies have shown that prolonged stays at home can raise other concerns, such as caring for family members and coping with stress, as well as physical (e.g. poor ergonomics or a work station) and mental health problems [4]. In fact, PhD students often report experiencing a sense of isolation during their training even in normal times, [22] which is likely exacerbated during COVID-19 [23, 24]. Analysing specific responses, it was apparent that many students suffer from mental health problems and poor emotional support (as highlighted in theme 6) during the pandemic, and that those who had supervisors that offer encouragement and sympathy fair better.

### Students with caretaking responsibilities

Recent commentaries suggested that students with caretaking responsibilities (e.g. childcare) may be particularly effected during COVID-19 [25, 26]. We did not find any differences in either sex or caretaking responsibilities between the two groups of students in Sweden. However, this finding should be interpreted with caution when interpolating to other countries, as Scandinavia has beneficial regulations for parents (e.g. paid parental leave for an extended period of time, paid childcare, paid time-off to care for sick children etc.). Nonetheless, several students from both groups highlighted specific challenges posed by caretaking responsibilities:

*From a respondent in group 1: “…I was pregnant at the time, in [the] UK pregnant women were recommended to self-isolate but this was not the case in Sweden and it was stressful that my boss would not let me work from home until it was demanded by the university as a whole”*.

*“[I would like] A better understanding of the problems with work life during the spring of the pandemic, when you have small children that are forced to stay at home due to sniffles.”*^1^ (student in group 1)

*“…understand that tasks take more time during a pandemic, especially … having to cope with family responsibilities.”* (student in group 2).

We believe this highlights the question of caretaking responsibilities as an important consideration.

### The clock is ticking – time delays and possible extension to PhD programs

PhD students are a vulnerable group due to financial and time-constraints imposed by their educational and research program. A common theme among students (particularly in group 2) was that they requested an extension of their PhDs, given delays caused by the pandemic. This may be particularly relevant for PhD students in the field of biomedicine and medicine, as these require physical presence in the lab and/or clinic to conduct their research, as also recently highlighted by several editorials [27–29]. An important consideration is where the funding for such an extension should come from. Many PhD students are funded by their supervisor’s external research grants. One approach could be that the university or the government provides the necessary funds to extend PhD programs. It may be important to consider that PhD programs in other countries (not Sweden) rely financially on graduate student tuition fees, particularly from international students. Thus, the pandemic poses a significant economic burden on universities, which may in turn affect the educational program [30].

### Limitations

The doctoral programs in Sweden/Scandinavia differ from many other countries. PhD studies are free of charge and students are entitled to generous parental leave and childcare facilities, rendering students less vulnerable compared to students in other countries. Sweden also had very few COVID-19 lockdown regulations and restrictions, which is unique world-wide, and even separate from other Scandinavian countries [31]. The data in this study should be interpreted taking this into consideration.

Response rates were difficult to assess as the survey was distributed through the administrative offices. In addition, it is unclear if the respondents encompass a representative sample of PhD students in the fields of biomedicine and medicine. Hence, our analysis is only based on submitted responses, and may thus not be generalized to all PhD students.

## Conclusions

The majority of students in the survey reported that the quality of their supervision was either improved or unaffected by the COVID-19 pandemic (group 1). However, nearly a third of students (27 %) felt that their supervision/mentorship had worsened (group 2). It is important for the scientific community to learn from this, to provide the best supervision possible in these difficult circumstances. The data suggests that more frequent online meetings and alternative communication platforms (email/messages and telephone) proved helpful. In addition, supervisors should ensure that the students receive emotional support during challenging times. Based on specific comments, some students would also benefit from an extension of their PhD programs due to delays caused by the pandemic.

## Data Availability

The datasets used and/or analysed during the current study are available from the corresponding authors on reasonable request.

## Abbreviations

SARS-CoV-2: Severe acute respiratory syndrome coronavirus 2
COVID-19: Coronavirus disease 2019
WHO: World Health Organization
N/A: Not applicable

## Footnotes

1 In Sweden, childcare facilities have been open during the pandemic, but children are not allowed to attend if showing any signs of illness and thus even a minor “sniffle” that would normally allow the child to addend pre-school/school meant that they needed to be at home.
